# 11-Hydroxyeicosatetraenoics induces cellular hypertrophy in an enantioselective manner

**DOI:** 10.3389/fphar.2024.1438567

**Published:** 2024-08-12

**Authors:** Sara A. Helal, Ahmed A. El-Sherbeni, Ayman O. S. El-Kadi

**Affiliations:** ^1^ Faculty of Pharmacy and Pharmaceutical Sciences, University of Alberta, Edmonton, AB, Canada; ^2^ Department of Biochemistry, Faculty of Pharmacy, Tanta University, Tanta, Egypt; ^3^ Department of Clinical Pharmacy, Faculty of Pharmacy, Tanta University, Tanta, Egypt

**Keywords:** arachidonic acid, cytochrome P450, hydroxyeicosatertraenoic acid, cellular hypertrophy, enantiomers

## Abstract

**Background:**

R/S enantiomers of 11-hydroxyeicosatertraenoic acid (11-HETE) are formed from arachidonic acid by enzymatic and non-enzymatic pathways. 11-HETE is predominately formed by the cytochrome P450 1B1 (CYP1B1). The role of CYP1B1 in the development of cardiovascular diseases is well established.

**Objectives:**

This study aimed to assess the cellular hypertrophic effect of 11-HETE enantiomers in human RL-14 cardiomyocyte cell line and to examine their association with CYP1B1 levels.

**Methods:**

Human fetal ventricular cardiomyocyte, RL-14 cells, were treated with 20 µM (R) or (S) 11-HETE for 24 h. Thereafter, cellular hypertrophic markers and cell size were then determined using real-time polymerase chain reaction (RT-PCR) and phase-contrast imaging, respectively. The mRNA and protein levels of selected CYPs were determined using RT-PCR and Western blot, respectively. In addition, we examined the effect of (R) and (S) 11-HETE on CYP1B1 catalytic activity using human recombinant CYP1B1 and human liver microsomes.

**Results:**

Both (R) and (S) 11-HETE induced cellular hypertrophic markers and cell surface area in RL-14 cells. Both enantiomers significantly upregulated CYP1B1, CYP1A1, CYP4F2, and CYP4A11 at the mRNA and protein levels, however, the effect of the S-enantiomer was more pronounced. Furthermore, 11(S)-HETE increased the mRNA and protein levels of CYP2J and CYP4F2, whereas 11(R)-HETE increased only CYP4F2. Only 11(S)-HETE significantly increased the catalytic activity of CYP1B1 in recombinant human CYP1B1, suggesting allosteric activation in an enantioselective manner.

**Conclusion:**

Our study provides the first evidence that 11-HETE can induce cellular hypertrophy in RL-14 cells via the increase in CYP1B1 mRNA, protein, and activity levels.

## 1 Introduction

Cardiac hypertrophy (CH) is a reversible response of the heart to various stress conditions, such as hypertension and chronic heart valve diseases. If left untreated, CH develops into more serious and irreversible heart failure (Nakamura and Sadoshima, 2018). It is characterized by the enlargement of the heart muscle cells and the thickening of the walls of the ventricles. CH can be categorized into two types: physiological and pathological. While CH typically begins as an adaptive response to the stimuli, it has a significant propensity to progress to heart failure when it becomes chronic and persistent (Shimizu and Minamino, 2016). Several studies have demonstrated the role of arachidonic acid (AA) metabolites in the pathogenesis of many cardiovascular diseases (CVD) including CH especially midchain hydroxyeicosatetraenoic acids (HETEs) (mainly 5-, 12-, and 15 HETEs) (Maayah and El-Kadi, 2016; Zhou et al., 2021; Hidayat et al., 2023a; Isse et al., 2023b; Helal et al., 2023).

It is well known that HETEs existing in biological systems are typically a combination of R and S configurations (Isse et al., 2023a). Interestingly 11-HETE, one of the midchain HETEs, can be generated either enzymatically or via non-enzymatic oxidation of AA. It can be produced by an enzymatic process, exclusively in the R-configuration, as a by-product of prostaglandin biosynthesis via the cyclooxygenase-1 (COX-1) and COX-2 enzymes (Xiao et al., 1997). Additionally, incubating rat liver microsome with AA produced 11(R)- and 11(S)-HETE via cytochrome P450 in NADPH-dependent metabolism with the R-enantiomer being more predominant than the S-enantiomer (Capdevila et al., 1986). Furthermore, cultured rat aorta smooth muscle cells synthesize both prostacyclin and significant quantities of 11- and 15-HETE via the COX pathway in response to AA or physiological stimuli such as thrombin (Bailey et al., 1983). The non-enzymatic synthesis of 11-HETE, as well as 8 and 9-HETE, occurs due to the free radical oxidation of AA (Feugray et al., 2022). The elevated plasma level of 11-HETE has been identified as a marker of lipid peroxidation and may suggest heightened oxidative stress and an increase in reactive oxygen species (Austin Pickens et al., 2019).

Each HETE enantiomer can exhibit distinct biological or pathophysiological effects. As shown previously in our laboratory, some HETE enantiomers may exert more pronounced effects on the induction of cardiac hypertrophy compared to the other enantiomers (Shoieb and El-Kadi, 2018; Hidayat et al., 2023b; Isse et al., 2023b). Since COX and cytochrome P450 (CYP) enzymes produce mainly 11(R) HETE and the levels of 11(S)-HETE are higher than those of 11(R)-HETE in isolated human plasma and serum, it was necessary to study the potential involvement of 11-HETE enantiomers in the process of CH.

## 2 Materials and methods

### 2.1 Materials

11-HETE (R and S) enantiomers were purchased from Cayman Chemical (Ann Arbor, MI, United States). Dulbecco’s Modified Eagle’s Medium/F-12 (DMEM/F-12) and trypsin were obtained from Gibco, Life Technologies (Grand Island, NY, United States). TRIzol reagent was an Invitrogen brand (Thermo Fisher Scientific, Carlsbad, CA). High-Capacity cDNA Reverse Transcription kit and SYBR^®^ Green PCR Master Mix were both obtained from Applied Biosystems (Foster City, CA, United States). According to the previously published sequences, Integrated DNA Technologies (Coralville, IA, United States) formulated the real-time polymerase chain reaction (RT-PCR) primers. Trans-Blot Turbo RTA Transfer Kit and 2X Laemmli Sample Buffer were purchased from Bio-Rad Laboratories (Hercules, CA, United States). Recombinant monoclonal CYP1B1 antibody (ab185954), CYP4A11 (ab3573), CYP4F2 (ab230709), and glyceraldehyde-3-phosphate dehydrogenase (ab8245) mouse monoclonal antibody was purchased from Abcam (Toronto, ON). CYP2J antibody (ABS1605) was obtained from Millipore Sigma (Burlington, MA, United States). Both the anti-mouse and the anti-rabbit IgG HRP-linked secondary antibodies were from Cell Signaling (Massachusetts, United States). Chemiluminescence Western blotting detection reagent (ECL) was purchased from GE Healthcare Life Sciences (Pittsburgh, PA, United States). Resorufin, 7-ethoxy resorufin (7-ER), nicotinamide adenine dinucleotide phosphate (NADPH) tetrasodium salt, and fetal bovine serum were purchased from Sigma Chemical Co (St. Louis, MO, United States). ProLong Gold Antifade, 4′,6-diamidino-2-phenylindole (DAPI), Alexa Fluor 488 Conjugate, and wheat germ agglutinin were obtained from Thermo Fisher Scientific (Edmonton, Canada). Human recombinant CYP1B1 supersomes supplemented with NADPH–cytochrome P450-oxidoreductase were obtained from (Gen test, MA, United States). Human liver microsomes (InVitroCYP™) were purchased from BioIVT (Hicksville, NY, United States). All other chemicals and reagents used in the experiments were purchased from Fisher Scientific Company (Toronto, ON).

### 2.2 Cell culture

Human fetal ventricular cardiomyocyte (RL-14) cells (Patent Deposit Designation No. PTA-1499) were purchased from the American Type Culture Collection (ATCC) (Manassas, VA, United States). RL-14 cells were grown in DMEM/F-12 with phenol red that is supplemented with 12.5% fetal bovine serum, 20 μM l-glutamine, 100 IU/ml penicillin G, and 100 μg/ml streptomycin. Cells were grown in 75 cm^2^ tissue culture flasks at 37°C under a 5% CO_2_ humidified environment. Each 75-cm^2^ flask had an average of 7 × 10^6^ cells. For seeding, each well contained an average of 9.8 × 10^5^ cells in the 6-well plate, an average of 3.5 × 10^5^ cells in the 12-well plate, and an average of 1.8 × 10^5^ cells in the 48-well plate. Cells were cultured in the complete media until they achieved a confluency state suitable for platting.

### 2.3 Chemical treatments

The cells in the control group were treated with the vehicle [serum-free DMEM/F-12 containing 0.5% dimethylsulfoxide (DMSO)]. The other groups were treated by adding 20 μM (R) or (S) 11-HETE to the serum free media (SFM) for 24 h. Both (R) and (S) 11-HETE were supplied as a stock solution in DMSO and were stored at −20°C until use. DMSO concentration didn’t exceed 0.5% in the treated groups during all the performed experiments.

### 2.4 Measurement of cell viability

Cell viability test was determined by using the 3-(4,5-dimethylthiazol-2-yl)-2,5-diphenyl-2H-tetrazolium bromide (MTT) assay which measures the ability of the living cells to reduce the yellow tetrazolium salt to its water-insoluble purple formazan crystals. The optical density of the formazan crystals reflects the population of living cells. Cells were plated in a 48-well plate under 37°C temperature and 5% CO_2_ humidified condition until sufficient confluency was achieved. Then the cells were treated with 2.5, 5, 10, and 20 μM of either (R) or (S) 11-HETE for 24 h. The media containing the treatment was discarded and 100 μL of MTT reagent (1.2 mM) dissolved in SFM was added and incubated with the cells at 37°C. After incubation for 2 h, the medium was discarded and 200 μL of DMSO were added to solubilize the formed formazan crystals. Synergy™ H1 Hybrid Multi-Mode Reader (BioTek Instruments; Winooski, VT, United States) was used to measure the color intensity at a wavelength of 570 nm.

### 2.5 Measurement of cell surface area

RL-14 cells were plated in a 6-well plate and treated with 20 μM of either (R) or (S) 11-HETE for 24 h. After that, cells were washed with 1x PBS (pH 7.4) 3 times and fixed with 4% paraformaldehyde for 15 min at 4°C. Then 10 μg/ml of wheat germ agglutinin, Alexa Fluor 488 conjugate was added, and the plates were incubated for 2 h in a dark place. The plates were washed again with 1x PBS (pH 7.4) 3 times each for 5 min using a shaker. Thereafter, the coverslips that have the stained cells were put on a glass slide with ProLong antifade reagent with DAPI. The slides were then imaged by an inverted microscope using the ×20 objective lens as described previously (Alammari et al., 2023) and the surface area was measured using Zeiss AxioVision Software (Carl Zeiss Imaging Solutions, version 4.8). Sixty-five individual cells from each group were included in the analysis.

### 2.6 RNA extraction and cDNA synthesis

RNA extraction and cDNA synthesis were performed on the (R) and (S) 11-HETE-treated RL-14 cells according to the method described previously (Shoieb and El-Kadi, 2020). In brief, cells were plated in 12-well plates and treated with 20 μM (R) and (S) 11-HETE for 24 h. Thereafter, the total RNA was isolated with TRIzol reagent, and the concentration was determined by measuring the absorbance at 260 nm. The RNA purity was determined by measuring the 260/280 ratio (>1.8). The first strand of cDNA was performed according to the manufacturer’s instructions by mixing 1.25 µg of the total RNA isolated from each sample with high-capacity cDNA reverse transcription reagents (Applied Biosystems). Finally, the reaction mixture was inserted in a thermocycler and underwent the following conditions: 25°C for 10 min, 37°C for 120 min, 85°C for 5 min, and at last it was cooled to 4°C.

### 2.7 Real-time polymerase chain reaction (RT-PCR) for quantification of mRNA gene expression

The mRNA gene expression was quantified in a 384-well optical reaction plate using Applied Biosystems Quant Studio 5 RT-PCR System. Each 20 µL of the reaction mixture contains equal volumes of forward and reverse primers (0.04 µL) with 20 nM final concentration of each, 8.92 µL of nuclease-free water, 10 µL SYBR Green Universal Master Mix, and 1 µL of the cDNA sample. Thermocycling conditions were as described previously (Shoieb et al., 2022) [initiation at 95°C for 10 min followed by 40 cycles of denaturation (95°C, 15 s) and combined annealing/extension (60°C, 60 s)]. The sequences of the human primers used in this study are listed in Table 1. Analysis of the RT-PCR data for the genes of interest and the reference gene (β-actin) was carried out using the relative gene expression (i.e., ΔΔ CT) method, as previously reported (Livak and Schmittgen, 2001).

**TABLE 1:** Primer sequences used for RT- PCR reactions.

Genes	Forward primer	Reverse primer
ANP	CAA​CGC​AGA​CCT​GAT​GGA​TTT	AGC​CCC​CGC​TTC​TTC​ATT​C
α-MHC	GCC​CTT​TGA​CAT​TCG​CAC​TG	GGT​TTC​AGC​AAT​GAC​CTT​GCC
β-MHC	TCA​CCA​ACA​ACC​CCT​ACG​ATT	CTC​CTC​AGC​GTC​ATC​AAT​GGA
ACTA-1	AGG​TCA​TCA​CCA​TCG​GCA​ACG​A	GCT​GTT​GTA​GGT​GGT​CTC​GTG​A
BNP	CAG​AAG​CTG​CTG​GAG​CTG​ATA​AG	TGT​AGG​GCC​TTG​GTC​CTT​TG
1B1	TTC​GGC​CAC​TAC​TCG​GAG​C	AAG​AAG​TTG​CGC​ATC​ATG​CTG
1A1	CTATCTGGGCTGTGGGCA	CTG​GCT​CAA​GCA​CAA​CTT​GG
4A11	CCA​TCC​CCA​TTG​CAC​GAC​TT	CAG​GTA​CAG​AAG​CAG​GTA​GGG
4F11	CAT​CTC​CCG​ATG​TTG​CAC​G	TCT​CTT​GGT​CGA​AAC​GGA​AGG
4F2	GAG​GGT​AGT​GCC​TGT​TTG​GAT	CAG​GAG​GAT​CTC​ATG​GTG​TCT​T
2J2	GAG​CTT​AGA​GGA​ACG​CAT​TCA​G	GAA​ATG​AGG​GTC​AAA​AGG​CTG​T
2E1	ATG​TCT​GCC​CTC​GGA​GTC​A	CGA​TGA​TGG​GAA​GCG​GGA​AA
2C8	CAT​TAC​TGA​CTT​CCG​TGC​TAC​AT	CTC​CTG​CAC​AAA​TTC​GTT​TTC
β-actin	CTGGCACCCAGCACAATG	GCC​GAT​CCA​CAC​GGA​GTA​CT

### 2.8 Protein extraction from RL-14 cells and western blot analysis

Protein extraction from the cells and Western blot analysis were performed as previously described by Shoieb and El-Kadi (2020). In brief, Rl-14 cells were grown in 6-well plates and incubated with 20 μM (R) or (S) 11-HETE for 24 h. Thereafter, the cell lysates were collected using 100 μL from the lysis buffer containing 50 mM HEPES, 1.5 mM magnesium chloride, 0.5 M sodium chloride, 10% (v/v) glycerol, 1 mM EDTA, 1% Triton X-100, and 5 μL/ml protease inhibitor cocktail. Subsequently, the Lowry assay was done to determine the concentration of the protein using bovine serum albumin as a reference standard (Lowry et al., 1951).

Western blot analysis was performed by separating 100 μg of the total cell lysate by 10% sodium dodecyl sulfate-polyacrylamide gel electrophoresis (SDS–PAGE). The separated proteins were transferred to polyvinylidene difluoride membranes and were incubated with the specific primary antibody for the desired protein overnight at 4°C. The membranes were then incubated with secondary antibodies (anti-rabbit IgG HRP-linked secondary antibodies or anti-mouse IgG HRP-linked secondary antibodies) in a blocking solution for 45 min at room temperature. The protein bands were finally visualized after the addition of ECL prime Western blot detection reagent using the enhanced chemiluminescence method and the ChemiDoc Imaging System (Bio-Rad Laboratories; CA, United States). The band’s signals were quantified relative to the signals obtained for the Glyceraldehyde-3-phosphate dehydrogenase (GAPDH) protein (loading control), using the Image Laboratory Software, version 6.1 (Bio-Rad Laboratories, Hercules, CA).

### 2.9 Effect of 11-HETE enantiomers on human recombinant CYP1B1 enzymatic activities

The rates of the O-dealkylation of 7-ethoxyresorufin catalyzed by recombinant human CYP1B1 were measured using the ethoxyresorufin-O-deethylase (EROD) assay. The measurements were conducted in the absence or presence of either (R) or (S) 11-HETE. The assay was done using a white 96-well microplate. Different concentrations of 7-ER (7-ethoxyresorufin; final concentration of 0, 5, 10, 20, 40, and 100 nM) were subjected to incubation with a reaction mixture containing 100 mM potassium phosphate (pH 7.4) buffer supplemented with 5 mM magnesium chloride hexahydrate and 1 pmol of human recombinant CYP1B1 enzyme. After that, various concentrations (final concentration of 0, 0.5, 2.5, 10, and 40 nM) of either (R) or (S) 11-HETE were added to the reaction mixture. Then 100 μL of this reaction mixture was added to each well of the 96-well plate followed by 100 μL of NADPH (2 mM) to start the reaction. The fluorescent reading of the plate related to the resorufin formation was measured by BioTek Synergy H1 Hybrid Reader (BioTek Instruments, Inc.) every min for 30 min. The signal was recorded with 550/585 nm excitation/emission wavelengths, respectively. A resorufin standard curve was prepared and used to calculate the amount of the resorufin formed. The rate of resorufin formation was plotted versus the concentration of 7-ER for each concentration of 11-HETE.

### 2.10 Effect of 11-HETE enantiomers on cytochrome P450 enzymatic activities in human liver microsomes

Incubation with human liver microsomes was performed to test the effect of either (R) or (S) 11-HETE on modulating the enzymatic activities of the CYP1B1 enzyme. Human liver microsomes pooled from 25 different individuals (0.1 mg/mL) were incubated with different concentrations of (R) or (S) 11-HETE (0, 10, 20, 40, and 100 nM). 2 μM of EROD was used as the substrate in the reaction that also contains 100 mM potassium phosphate buffer (pH 7.4) supplemented with 5 mM magnesium chloride hexahydrate. 100 μL of the reaction mixture was added to the wells of 96-well polystyrene microplates. To start the reaction, 100 μL of 1 mM NADPH was added to the reaction mixture in each well. A BioTek Synergy H1 Hybrid Reader (BioTek Instruments, Inc) was used to measure the fluorescent signal generated due to the formation of resorufin every minute for 30 min under 37°C at 550/585 nm excitation/emission wavelengths, respectively.

### 2.11 Statistical analysis

The results were represented as mean ± standard error of the mean (SEM). Data were analyzed using one-way analysis of variance (ANOVA) followed by a Tukey *post hoc* test for all experiments except the microsomal and supersome incubation experiments where we used Dunnett test. The result was considered significantly different when the *p*-value was less than 0.05. The rate of resorufin formation was plotted against 7-ER concentration and was fitted considering each replicate as an individual point to the Michalis-Menten model. All the Statistical analysis, the graph plotting, and the enzymology module were executed using GraphPad Prism software for Windows, version 8.4.3. (GraphPad Software, Inc. La Jolla, CA).

## 3 Results

### 3.1 Effect of (R) and (S) 11-HETE on cell viability

MTT assay was used to assess the cytotoxicity of the 11-HETE concentrations used. RL-14 cells were treated with 2.5, 5, 10, and 20 μM of (R) and (S) 11-HETE for 24 h. All the concentrations tested did not significantly alter the cell viability (depicted by the viability above 90%) when compared to the control (data not shown). As a result, we used the 20 μM concentration in all the subsequent experiments.

### 3.2 Effect of 11-HETE enantiomers on cellular hypertrophic markers in RL-14 cells

**FIGURE 1:**
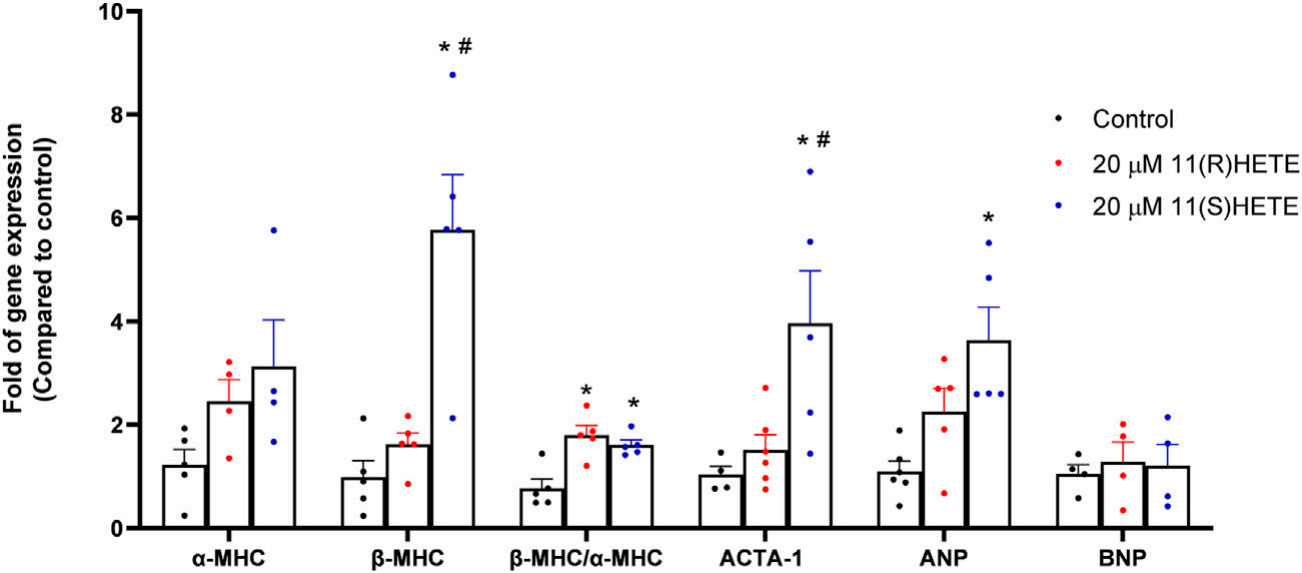
Effect of (R) and (S) 11-HETE on cellular hypertrophy in RL-14 cells. RL-14 cells were treated for 24 h with 20 μM of 11-HETE enantiomers; then, the mRNA levels of ANP, α-MHC, β-MHC, ACTA-1, and BNP were quantified using real-time PCR. The values represent mean ± SEM (n = 4–5). Data were analyzed using one-way ANOVA. *p < 0.05 significant compared to the control group, #*p* < 0.05 significant compared to the 11(R)-HETE treated group.

To evaluate the potential effect of 11-HETE enantiomers in inducing cellular hypertrophy, RL-14 cells were treated with 20 μM of either 11(R)-HETE or 11(S)-HETE for 24 h. Thereafter, the expressions of the cardiac hypertrophic markers such as atrial natriuretic peptide (ANP), α-myosin heavy chain (α-MHC), β-MHC, skeletal α-actin (ACTA-1), and brain natriuretic peptide (BNP) were measured using RT-PCR. Figure 1 shows that 11(S)-HETE significantly increased the cardiac hypertrophic markers: ANP, β-MHC, and β/α-MHC by 231%, 499%, and 107%, respectively. 11(R)-HETE significantly increased the cardiac hypertrophic marker; β/α-MHC by 132%. Furthermore, ACTA-1 gene expression was increased by 46% in the 11(R)-HETE-treated group and was significantly increased by 282% in the 11(S)-HETE-treated group compared to the control (Figure 1). Both β-MHC and ACTA-1 gene expression were significantly increased in the 11(S)- compared to the 11(R) HETE-treated group.

**FIGURE 2:**
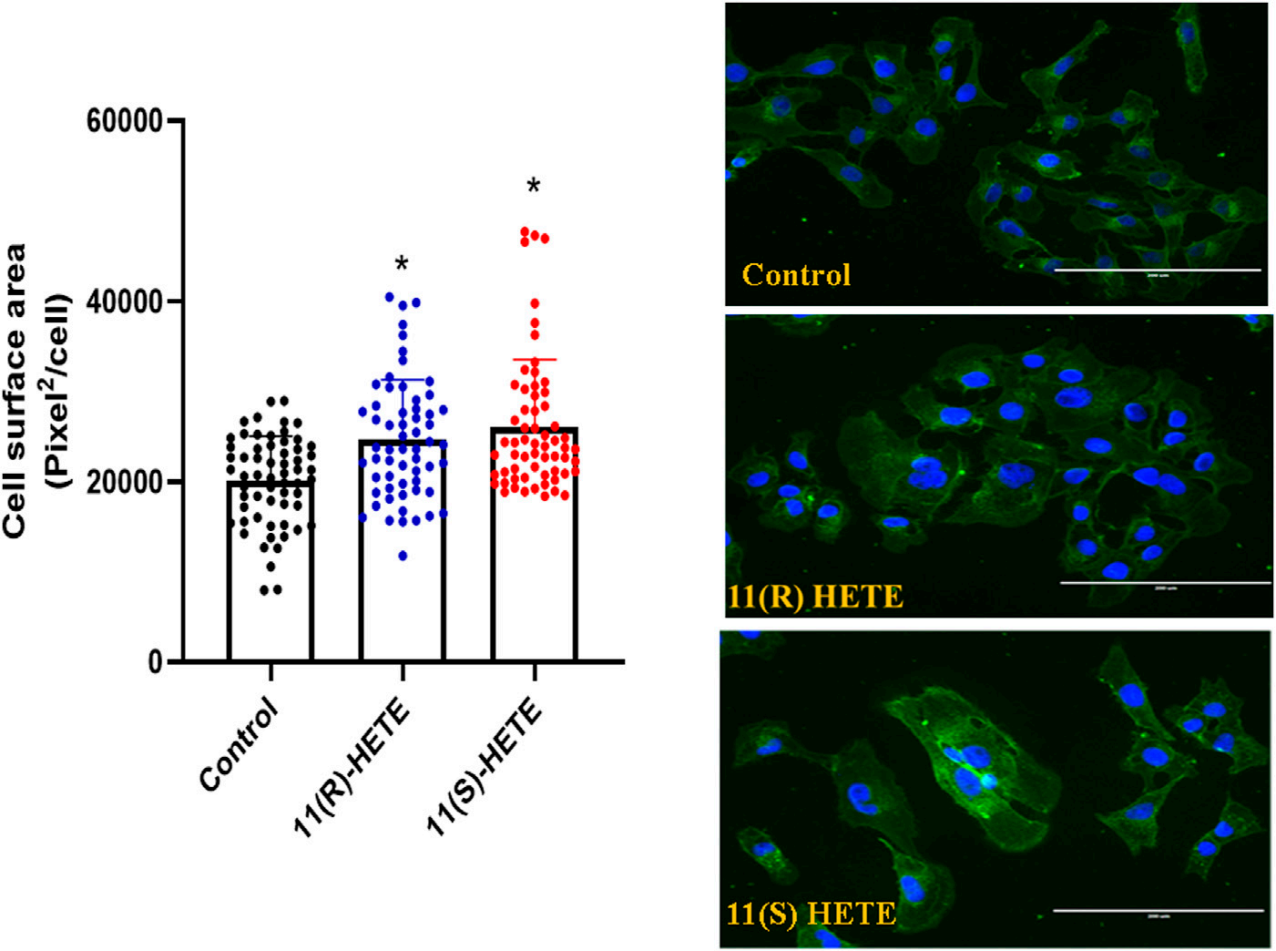
Effect of 11-HETE enantiomers on RL-14 cells surface area. RL-14 cells were treated with 20 µM of 11-HETE enantiomers for 24 h. Cell surface area was then determined by phase-contrast imaging using Zeiss Axio Observer Z1 inverted microscope using a ×20 objective lens. The values represent mean ± SEM (n = 65). Data were analyzed using one-way ANOVA. *p < 0.05 significant compared to the control group.

It was established that the increase in the hypertrophic markers is associated with the increase in the cell surface area. As shown in Figure 2, our results showed that treating the RL-14 cells with 20 μM of either (R) or (S)- 11 HETE significantly increased the cell surface area by 29% and 34% compared to the control, respectively.

### 3.3 Effect of 11-HETE enantiomers on CYP mRNA gene expression in RL-14 cells

**FIGURE 3:**
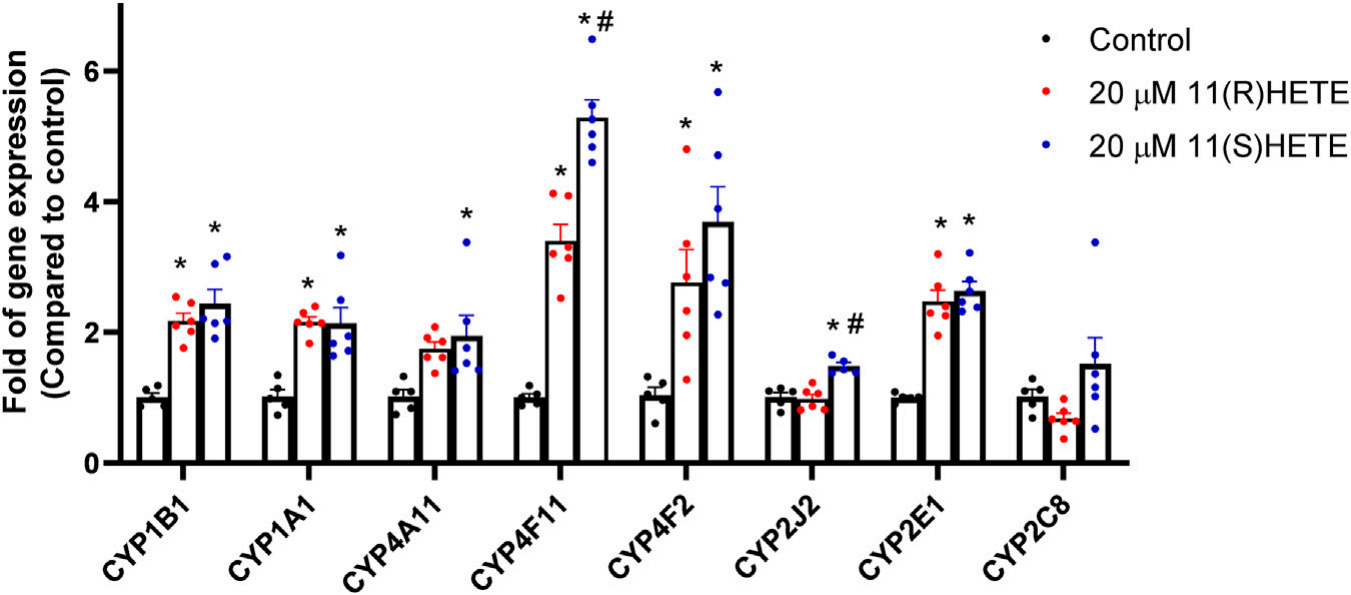
Effect of 11-HETE enantiomers on the CYPs gene expression. RL-14 cells were treated with 20 µM of 11-HETE enantiomers for 24 h. After that, CYP1B1, CYP1A1, CYP4A11, CYP4F11, CYP4F2, CYP2J2, CYP2E1, and CYP2C8 gene expression were quantified using real-time PCR and normalized to ß-actin. The values represent mean ± SEM (n = 5–6). Data were analyzed using one-way ANOVA. *p < 0.05 significant compared to the control group, #*p* < 0.05 significant compared to the 11(R)-HETE treated group.

To examine the effect of 11-HETE enantiomers on CYP enzymes, RL-14 cells were treated with 20 μM (R) or (S) 11-HETE for 24 h. Thereafter, CYP1B1, CYP1A1, CYP4A11, CYP4F11, CYP4F2, CYP2J2, CYP2E1 and CYP2C8 mRNA were determined using RT-PCR. The CYP1B1, CYP1A1, CYP4A11, CYP4F11 and CYP4F2 mRNA were significantly increased in the cells treated with 11(R)-HETE by 116%, 112%, 70%, 238% and 167%, respectively, compared to the control group. Similarly, the 11(S)-HETE-treated group showed a significant increase in the gene expression of the same enzymes by 142%, 109%, 90%, 416% and 257% respectively, compared to the control (Figure 3). Albeit both (R) and (S) enantiomers have significantly increased the CYP2E1 mRNA gene expression by 146% and 163% respectively compared to the control group, only 11(S)-HETE increased the CYP2J2 mRNA gene expression by 47%.

### 3.4 Effect of 11-HETE enantiomers on the protein level of CYP enzymes in RL-14 cells

**FIGURE 4:**
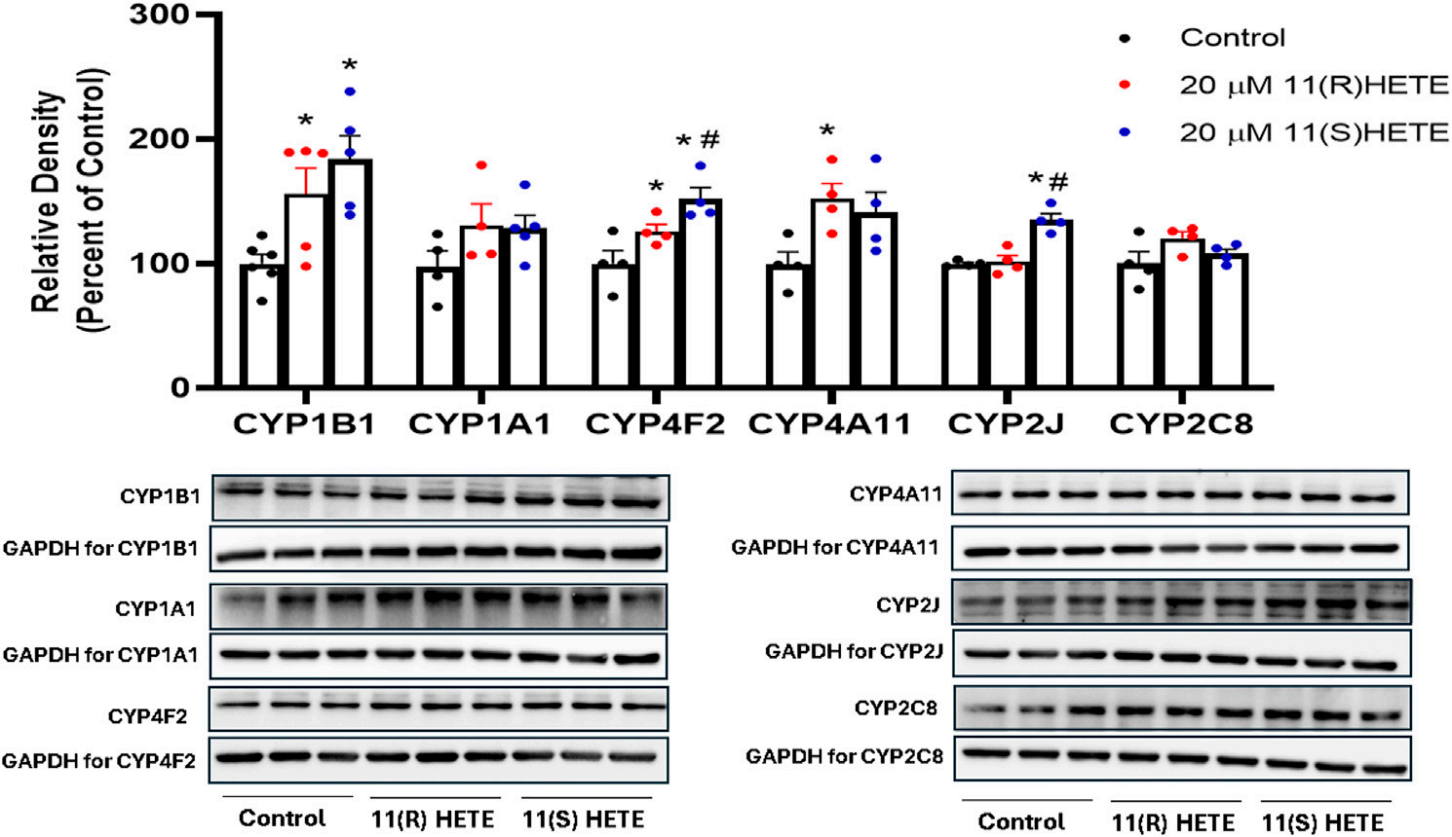
Effect of 11-HETE enantiomers on CYPs protein level. RL-14 cells were treated with 20 µM of 11-HETE enantiomers for 24 h. After that, cell lysates were harvested, and CYP1B1, CYP1A1, CYP4F2, CYP4A11, CYP2J, and CYP2C8 protein levels were determined using Western blot analysis. The values represent mean ± SEM (n = 4–5). Data were analyzed using one-way ANOVA. *p < 0.05 significant compared to the control group, #*p* < 0.05 significant compared to the 11(R)-HETE treated group.

It was essential to assess the protein levels of the CYP enzymes of interest since the mRNA expression may not consistently align with the levels of these enzymes in terms of protein expression. The protein level of CYP1B1, CYP4F2, and CYP4A11 in the cells treated with 11(S-) HETE showed a significant increase by 186%, 153%, and 152%, respectively, compared to the control. While the 11(R)-HETE-treated cells did not affect the protein level to the same degree, it significantly increased the protein level of CYP1B1, CYP4F2, and CYP4A11 by 156%, 126%, and 141%, respectively, compared to the control (Figure 4).

Interestingly, the CYP2J protein level was significantly increased in the cells treated with 11(S-HETE) enantiomers by 135%, compared to the control group. Regarding CYP2C8, the increase in the protein level was not significant for both enantiomers (Figure 4). There was an increase in the gene expression of CYP4F11 for both enantiomers, however, CYP4F11 protein level was below the detection limit.

### 3.5 Effect of 11-HETE enantiomers on recombinant human CYP1B1 enzyme activity

**FIGURE 5:**
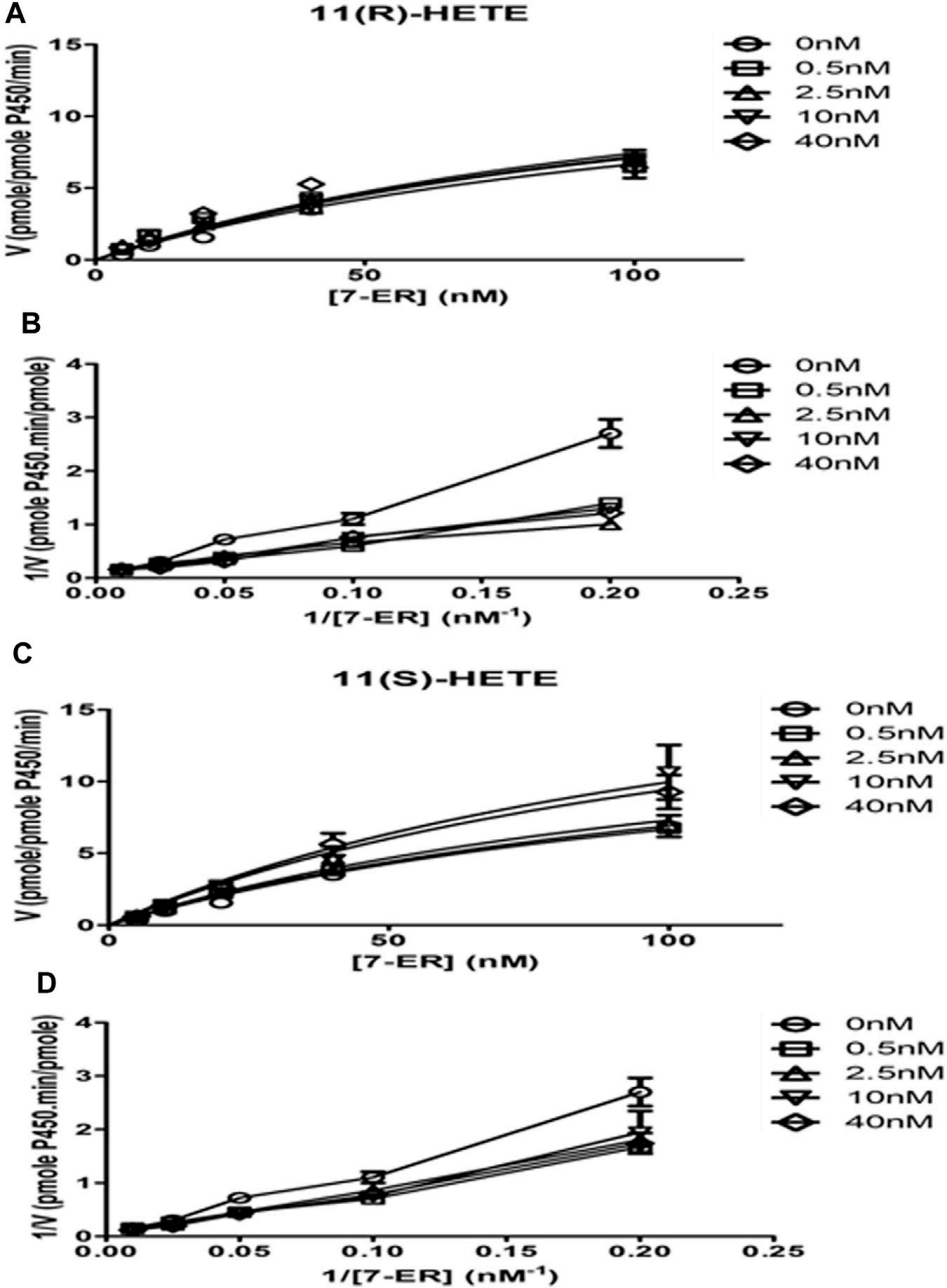
Effect of 11-HETE enantiomers on recombinant human CYP1B1 enzyme activity. **(A)** 11(S)-HETE allosterically activated CYP1B1 activity; **(C)** 11(R)-HETE did not affect CYP1B1 activity; **(B,D)** Lineweaver-Burk plots show intercepting lines for S and R enantiomers of 11-HETE. Using a white 96-well microplate, the reaction mixture containing both buffer and 1 pmol of recombinant human CYP1B1 was incubated with 0–100 nM of 7-ER. After that, 0, 0.5, 2.5, 10, and 40 nM of 11 (R) or (S)-HETE were added. Then, 100 μL of 2 mM NADPH was added to each well to start the reaction. The fluorescent signal related to resorufin formation was measured every minute for 30 min at 550/585 nm excitation/emission wavelengths using BioTek Synergy H1Hybrid reader. The quantity of formed resorufin was calculated by forming a standard curve of 0–200 nM resorufin dissolved in the same incubation buffer. Data displays mean ± SEM (n = 3).

The direct effect of R and S enantiomers of 11-HETE on rhCYP1B1 catalytic activity was assessed using rhCYP1B1-mediated EROD. The rate of resorufin formation (V) by rhCYP1B1 with various concentrations of 7-ER co-incubated with either S or R enantiomers of 11-HETE is shown in Figures 5A,C. 11(S)-HETE led to allosteric activation of CYP1B1 activity, causing a concentration-dependent increase in Vmax value, compared with control, by 1.03, 1.1, 1.5 and 1.4-fold for 0.5, 2.5, 10 and 40 nM of 11(S)-HETE, respectively (Table 2); whereas, 11(R)-HETE did not affect Vmax (Table 2). Km values of 7-ER hydrolysis of rhCYP1B1 did not change by either R or S enantiomers of 11-HETE; therefore, shared Km value was assumed in Michaelis-Menten model fitting, estimated to be 131.3 nM. The double reciprocal (Lineweaver-Burk) plots show intercepting lines for S and R enantiomers of 11-HETE, which in terms of allosteric interactions means changes in Vmax with no substantial effect on Km (Figures 5B,D).

**TABLE 2:** Best fit values for resorufin formation rate kinetics mediated by recombinant human CYP1B1.

	0 nM	0.5 nM	2.5 nM	10 nM	40 nM
11(R)-HETE					
Vmax	15.4 ± 2.2	16.4 ± 2.7	16.6 ± 2.8	16.6 ± 2.7	17.2 ± 2.8
Km	131.3 ± 27.7				
11(S)-HETE					
Vmax	15.4 ± 2.2	16.0 ± 2.7	16.9 ± 2.8	23.0 ± 3.3*	21.8 ± 3.1*
Km	131.3 ± 27.7				

The mean ± SD for Vmax and Km parameters for CYP1B1 activity in the absence and presence of 11-HETE enantiomers (n > 3). The enzyme kinetics were determined from best fit using the Enzyme kinetic module in GraphPad Prism. Vmax, maximum velocity; Km, the substrate concentration that provides the enzyme to achieve half Vmax. *p < 0.05 significant compared to the control group.

### 3.6 Effect of 11-HETE enantiomers on CYP1B1 activity in the human liver microsomes

**FIGURE 6:**
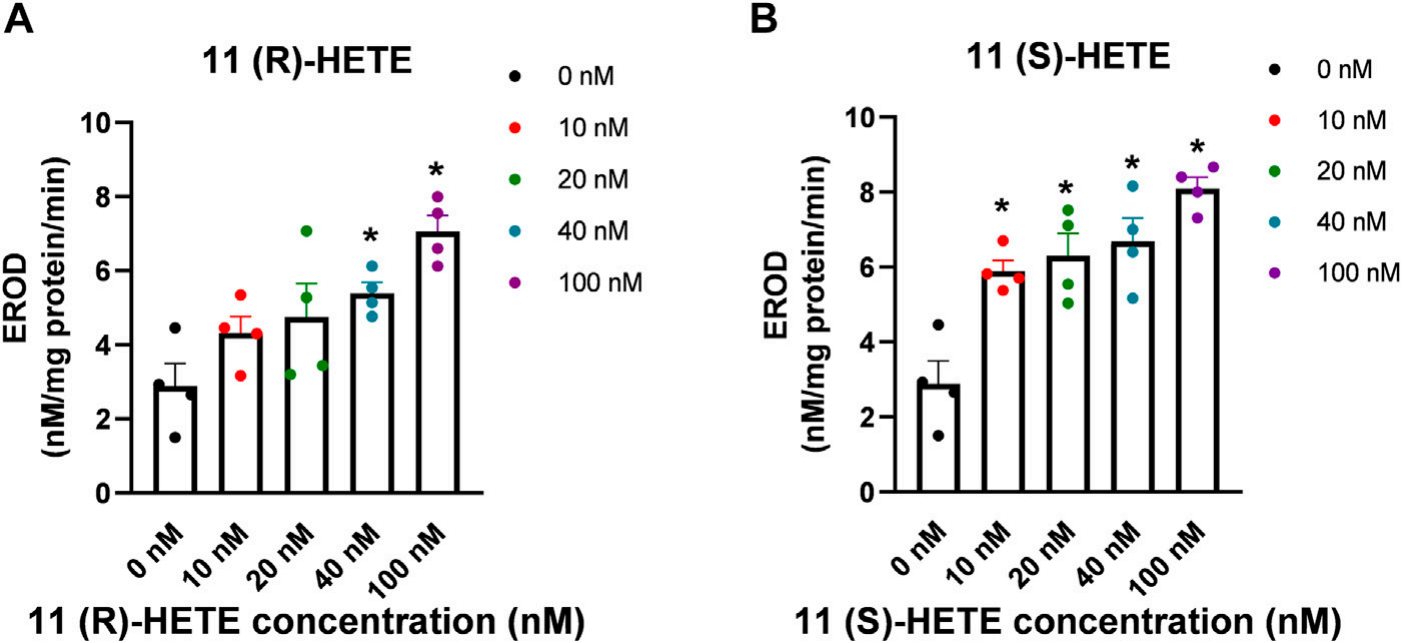
Effect of 11-HETE enantiomers on CYP1B1 activity in the human liver microsomes. **(A)** 11(R)-HETE effect on CYP1B1 activity in the human liver microsomes; **(B)** 11(S)-HETE effect on CYP1B1 activity in the human liver microsomes. 11-HETE enantiomers increase EROD activity in human liver microsomes. Human liver microsomes were used in a concentration of 0.1 mg/mL in the absence and presence of varying concentrations of 11-HETE enantiomers. In addition, 2 μM of 7-ER was used as a substrate. The reaction was initiated by adding 100 μL of 1 mM NADPH, and the fluorescent signal related to the formation of resorufin was determined. The values represent mean ± SEM (n = 4). Data were analyzed using one-way ANOVA. *p < 0.05 significant compared to the control group.

To further confirm the results obtained from rhCYP1B1, we have tested the possible effect of both 11-HETE enantiomers on the catalytic activity of CYP1B1 using EROD assay in the human liver microsomes. We used fixed concentrations of the substrate and varying concentrations of either 11(R) or 11(S) HETE (0, 10, 20, 40, and 100 nM). As shown in Figure 6, the results showed that incubation of human liver microsomes with increasing concentrations of 11(S)-HETE was associated with a concentration-dependent increase in the EROD formation rate when compared to the control group. 11(S)-HETE showed a stronger effect than 11(R)-HETE. A significant increase in the catalyzed EROD activity to 107%, 119%, 136%, and 183% was observed for the 10, 20, 40, and 100 nM of the 11(S)HETE compared to the control, respectively. Similarly, the concentrations of 40 and 100 nM 11(R)-HETE showed a significant increase to 87% and 145%, respectively (Figure 6).

## 4 Discussion

In the current study, the effect of (R) and (S) enantiomers of 11-HETE was found to induce hypertrophy in human cardiomyocytes. This was associated with an increase in CYP1B1 activity through direct activation of CYP1B1 and upregulation of CYP1B1 levels, which is known to mediate the formation of cardiotoxic metabolites, midchain HETEs. 11(S)-HETE was found to be more potent, compared with 11(R)-HETE, concerning the induction of hypertrophy, as well as the increase in CYP1B1. The catalytic activity of CYP1B1 in recombinant human CYP1B1 was also significantly increased by the (S) enantiomer, indicating allosteric activation. Since 11(S)-HETE is mainly produced via the interaction of AA with reactive oxygen species, these results link oxidative stress with the induction of CYP1B1 in the heart and the development of cellular hypertrophy.

Arachidonic acid is hydroxylated, *in vivo*, to 11 different hydroxy-arachidonic acids; HETEs. 5-, 8-, 9-, 11-, 12-, 15-, 16-, 17-, 18-, 19-HETEs (except for 20-HETE) exist in two configurations (R and S enantiomers) (Helal et al., 2024). Previous studies in our laboratory have shown the enantioselective differences in the 19-, 16- and 17- HETEs. The S-enantiomer of 19-HETE exhibited a selective inhibition of the catalytic activity of the CYP1B1 enzyme (Shoieb et al., 2019) and protect against cellular hypertrophy induced by angiotensin II in RL-14 and H9c2 cell lines (Shoieb and El-Kadi, 2018). In adult human cardiomyocytes AC-16 cell lines, cellular hypertrophy was significantly induced by 17(S)-HETE and exhibited greater allosteric activation of human recombinant CYP1B1 enzyme compared to 17(R)-HETE (Isse et al., 2023b). In addition, the 16(R)-HETE enantiomer upregulated CYP1B1 gene and protein expression and significantly increased the cellular hypertrophy in RL-14 cells greater than its enantiomer, 16(S)-HETE (Hidayat et al., 2023a).

The contribution of 11-HETE in several pathological conditions has been previously reported. Elevated plasma levels of 11-HETE, reaching up to six times the normal level, were observed in patients with hyperplastic colon polyps and adenomas and this could be an early indicator of the progression of malignant tumors (Austin Pickens et al., 2019). In COVID-19 patients, lower AA levels and remarkably elevated levels of both 5- and 11-HETE were detected in the bronchoalveolar lavage fluid. This finding provides strong evidence that 11-HETE can mediate the innate immune response to COVID-19 and could play a crucial role in determining the outcome of the infection (Pérez et al., 2021). An increased plasma level of 11-HETE is indicative of elevated oxidative stress and enhanced levels of reactive oxygen species production (Guido et al., 1993). Another study showed that 11-HETE exhibited a positive correlation with body mass index, waist circumference, and elevated serum leptin levels in individuals with obesity (Pickens et al., 2017). The content of 11-HETE in the rats brain also showed a significant increase 72 h after middle cerebral artery occlusion in comparison to the normal brain (Usui et al., 1985). Previous reports also indicated an association between elevated baseline levels of 11-HETE and the subsequent occurrence of acute myocardial infarction. Furthermore, a positive correlation exists between the serum levels of this metabolite and the levels of certain inflammatory and cardiac biomarkers, including tumor necrosis factor-α and pro-BNP (Huang et al., 2020).

In the current study we determined the gene expression and the protein level of important CYP enzymes that are responsible for metabolizing AA; hydroxylases (CYP1B1, CYP1A1, CYP2E1, CYP4A11, and CYP4F) and epoxygenases (CYP2C8, CYP2J) after treating RL-14 cells with either (R) or (S) 11-HETE. These enzymes produce metabolic products with varying effects, which can either be protective or pathogenic (Alsaad et al., 2013). The epoxygenases create a range of regiospecific and stereospecific epoxides (5,6-, 8,9-, 11,12-, and 14,15-epoxyeicosatrienoic acids (EETs)), while the ω-hydroxylases generate subterminal and ω-terminal HETEs (Kroetz and Zeldin, 2002).

CYP1B1 enzyme is one of the hydroxylases that are constitutively expressed in numerous tissues, most importantly in the heart, and mainly contributes to the formation of midchain HETEs (Carrera et al., 2020). The upregulation of CYP1B1 and its cardiotoxic metabolites, midchain HETEs, is a prevalent feature in various diseases and conditions, including inflammation, cancer, and cardiac hypertrophy (Li et al., 2017). The relation between the level of CYP1B1 enzyme and the induction of cardiac hypertrophy due to the increase in the production of midchain HETEs has been well-established (Maayah et al., 2017). In the current study, the treatment of RL-14 cells with the S-enantiomer of 11-HETE significantly increased the gene expression and the protein level of CYP1B1. The effect of the R-enantiomer was not as strong as the S-enantiomer in the upregulation of the mRNA and protein level of the CYP1B1. Furthermore, there was a concentration-dependent CYP1B1 activation in the concentrations between 0–40 nm of (S) 11-HETE when performing the EROD assay using rhCYP1B1enzyme which suggests an allosteric activation of the enzyme. To further confirm the results, both enantiomers were incubated with human liver microsomes, and the EROD formation rate was determined in the presence of different concentrations of the 11-HETE. There was a significant gradual increase in the EROD formation rate, and the S-enantiomer effect was more pronounced than the R-enantiomer. These effects on the level and the activity of CYP1B1 were consistent with the previous studies in our laboratory which showed that both 16-HETE (Hidayat et al., 2023b) and 17-HETE (Isse et al., 2023b) allosterically activated the CYP1B1 enzyme in an enantioselective manner.

In addition to CYP1B1, the increase in other hydroxylases such as CYP1A1, CYP4A11, CYP4F11, CYP4F2 and CYP2E1 at the gene expression and protein level has been reported previously. Both CYP1B1 and CYP1A1 were reported to be upregulated in the hearts of rats treated with isoprenaline-induced cardiac hypertrophy (Zordoky et al., 2008). The presence of CYP1A1 mRNA has also been observed in the right ventricle and left atrium among patients with dilated cardiomyopathy (Thum and Borlak, 2000). CYP4A11 was reported to be upregulated by 2- to 3-fold in patients with hypertrophic left ventricles in comparison to control (Thum and Borlak, 2002). In addition, CYP4F showed high expression in patients with cardiovascular diseases (Chaudhary et al., 2009). Moreover, the increased expression of CYP1A1, CYP1B1, CYP4A11, and CYP4F leads to an elevation in the 20-HETE formation rate. Enhanced formation of 20-HETE was reported to be involved in hypertrophic cardiac remodeling and cardiac failure (Rocic and Schwartzman, 2018) and contributes to the exaggeration of ischemia-reperfusion injury (Chaudhary et al., 2009). Moreover, increased expression of CYP2E1 in the heart has various pathophysiological implications, encompassing heightened oxidative stress and apoptosis (Guan et al., 2019; Ren et al., 2019). Elevated levels of CYP2E1 have been observed in the ischemic and dilated human heart, as well as in the left ventricular tissue of spontaneously hypertensive rats (Thum and Borlak, 2002). There was also an upregulation in the expression of CYP2E1 in the hearts affected by dilated cardiomyopathy in cTnT^R141W^ transgenic mice (Zhang et al., 2011).

In addition to the increase in the CYP hydroxylases, there was also an increase in the mRNA expression of some epoxygenases mainly CYP2J2 and CYP2C8. Although the CYP2J protein level of the cells treated with 11(S)-HETE increased significantly compared to the control group, there was no significant increase in the protein level of CYP2C8. The mRNA expression of CYP2J2 in humans is abundant in the cardiovascular system and liver, with a predominant presence in the right ventricle of the heart (Solanki et al., 2018). CYP2J2 and CYP2C8 have been identified as the primary CYP isoforms expressed in healthy human hearts, with CYP2J2 mRNA levels significantly surpassing those of CYP2C8. While numerous studies have documented the biologically protective role of these CYP enzymes and their products, EETs (Wang et al., 2016; Xu et al., 2016), some research findings have demonstrated the contrary. In individuals with hypertrophic hearts, there was an observed elevation in the expression of CYP2J, impacting ventricular force and leading to an increase in the heart’s muscle mass (Tan et al., 2002). A fivefold increase in CYP2J has been documented in human hypertrophic hearts compared to assist device-supported hearts (Thum and Borlak, 2002). While CYP2J2 has been shown to contribute to enhanced postischemic functional recovery in mice, Wang et al. proposed the involvement of CYP2J in cocaine-induced cardiac toxicity (Wang et al., 2002). Some research justified this increase as a protective mechanism from the heart cells to overcome the deleterious effects caused due to hypertrophy.

## 5 Conclusion

To our knowledge this is the first study to investigate the cellular hypertrophic effect of 11-HETE enantiomers in RL-14 cells and the mechanisms involved. Both R and S enantiomers of the midchain 11-HETE could induce cellular hypertrophy in RL-14 cells. They significantly increased several hypertrophic markers as well as the protein level and the gene expression of various CYP enzymes. The S- enantiomer allosterically activates human recombinant CYP1B1 and both enantiomers significantly increased the EROD activity in human liver microsomes. The investigation of the role of high 11-HETE concentration in various cardiovascular diseases should be expanded, and strategies to inhibit its effect could be tested as potential therapeutic strategies.

## Data Availability

The raw data supporting the conclusions of this article will be made available by the authors, without undue reservation.

## Abbreviations

7-ER, 7-ethoxyresorufin; AA, arachidonic acid; ACTA-1, skeletal α-actin-1; ANP, atrial natriuretic peptide; BNP, brain natriuretic peptide; CH, cardiac hypertrophy; COX, cyclooxygenase; CYP, cytochrome P450; EET, epoxyeicosatrienoic acids; EROD, ethoxyresorufin-O-deethylase; HETE, hydroxyeicosatetraenoic; MHC, myosin heavy chain; MTT, 3-(4,5-dimethylthiazol-2-yl)-2,5-diphenyl-2H-tetrazolium bromide; RT-PCR, real-time polymerase chain reaction.
